# Hypoxia promotes pancreatic adenocarcinoma progression by stabilizing ID1 via TRIM21 suppression

**DOI:** 10.3389/fonc.2025.1616968

**Published:** 2025-08-21

**Authors:** Rui Cheng, Yuanjun Tang, Xuedi Cao, Zhanya Huang, Yunyun Guo, Renjing Jin, Yan Wang, Yang Liu, Lixiang Xue, Yuqing Wang

**Affiliations:** ^1^ Peking University Third Hospital Cancer Center, Department of Radiation Oncology, Peking University Third Hospital, Beijing, China; ^2^ Center of Basic Medical Research, Institute of Medical Innovation and Research, Peking University Third Hospital, Beijing, China; ^3^ Cancer Center of Peking University Third Hospital, Beijing, China; ^4^ Biobank of Peking University Third Hospital, Peking University Third Hospital, Beijing, China; ^5^ Beijing Key Laboratory for Interdisciplinary Research in Gastrointestinal Oncology, Peking University Third Hospital, Beijing, China

**Keywords:** hypoxia, pancreatic adenocarcinoma, Id1, TRIM21, ubiquitination

## Abstract

**Introduction:**

Pancreatic adenocarcinoma (PAAD) is a highly aggressive malignancy characterized by a profoundly hypoxic tumor microenvironment, which fosters tumor progression and confers resistance to therapy The oncogenic regulator ID1has been implicated in PAAD malignancy, however, the mechanisms underlying hypoxia-induced stabilization of ID1 and the role of ubiquitin-mediated degradation remain poorly understood. Elucidating these pathways is essential for identifying novel therapeutic targets for PAAD.

**Methods:**

In this study, we examined ID1 expression in PAAD tissues and cell lines using publicly available databases and in vitro models. We simulated hypoxic conditions to assess their effects on ID1 expression and tumor cell behaviors, including proliferation, migration, and invasion. Protein stability was investigated via cycloheximide chase, proteasome and autophagy inhibition, and ubiquitination assays. Mass spectrometry identified TRIM21 as an E3 ubiquitin ligase interacting with ID1. To investigate its regulatory role, we generated stable TRIM21 knockdown and overexpression pancreatic cancer cell lines. Finally, in vivo xenograft experiments were conducted to evaluate the impact of ID1 and TRIM21 on tumor growth.

**Results:**

ID1 was markedly overexpressed in PAAD tissues and cell lines, correlating with advanced tumor stage, metastasis, and reduced patient survival. Hypoxia elevated ID1 protein levels without significantly affecting its mRNA, suggesting post-translational stabilization. Mechanistic studies revealed that hypoxia inhibits ubiquitin-proteasome-mediated degradation of ID1 by downregulating TRIM21, an E3 ubiquitin ligase responsible for ID1 ubiquitination. TRIM21 knockdown restored ID1 levels and promoted tumor cell function, whereas TRIM21 overexpression suppressed these malignant phenotypes and mitigated hypoxia-induced aggressiveness. In vivo, ID1 silencing impeded, while TRIM21 knockdown accelerated, pancreatic tumor growth, confirming their opposing roles in tumor progression.

**Discussion:**

Our findings demonstrate that hypoxia drives pancreatic tumor progression by downregulating TRIM21, leading to stabilization of the oncogenic protein ID1. The TRIM21–ID1 axis emerges as a promising therapeutic target for PAAD, suggesting that restoring TRIM21-mediated ID1 degradation could counteract hypoxia-induced malignancy.

## Graphical Abstract

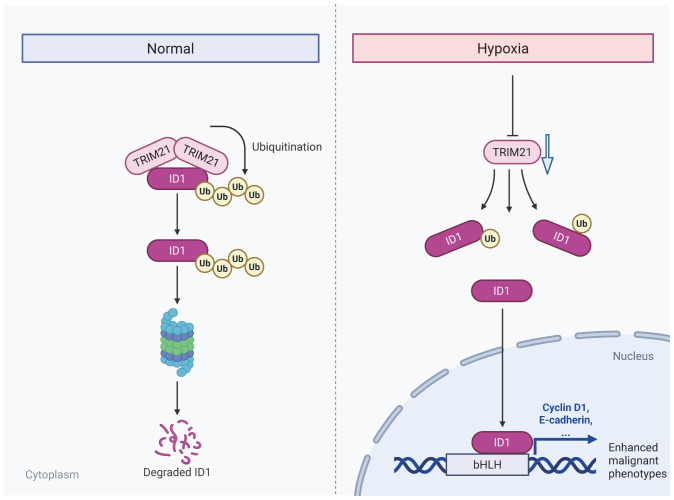

## 1 Introduction

Pancreatic adenocarcinoma, a highly aggressive malignancy originating from the ductal and acinar epithelium of the pancreas, represents one of the most lethal human cancers with a dismal 5-year survival rate of <10%. Its reputation as the “king of cancers” stems from the characteristic late diagnosis, rapid progression, and remarkable resistance to conventional therapies. While recent advances in neoadjuvant therapies and surgical techniques have improved outcomes for a subset of patients, complete surgical resection remains the only potentially curative option (1–4). However, pancreatic adenocarcinoma often frequently escapes early detection due to its insidious onset and tendency for early micrometastasis. Consequently, most patients are diagnosed with locally advanced or metastatic disease, rendering them ineligible for surgical resection. Although chemotherapy has improved patient prognosis to some extent, its efficacy remains limited, and drug resistance is commonly observed (5–7). This clinical challenge highlights the critical need to better understand the molecular mechanisms driving PAAD progression and to discover novel therapeutic targets.

The tumor microenvironment plays a critical regulatory role in cancer progression, especially during the transition from precancerous lesions to malignant tumors, by mediating stress signals that drive clonal selection and evolution (8). In pancreatic adenocarcinoma, a hallmark of its pathophysiology is a profoundly hypoxic microenvironment, resulting from the tumor’s high proliferative demand in combination with its dense desmoplastic stroma and poor vascularization (9–11). Hypoxia occurs in the majority of solid tumors and serves as a critical regulator of multiple oncogenic processes, including metabolic reprogramming, epithelial-mesenchymal transition (EMT), angiogenesis, and immune suppression (12–16). It also plays a key role in shaping the tumor microenvironment and modulating gene expression (17–20). However, the complete repertoire of hypoxia-responsive effectors and their precise mechanisms of action in PAAD pathogenesis remain incompletely understood.

ID1, a key member of the helix-loop-helix (HLH) family, acts as a dominant-negative regulator of basic HLH transcription by sequestering them in non-functional heterodimers. Beyond its well-established role in embryonic development and stem cell maintenance, ID1 has emerged as a critical mediator of oncogenic processes across multiple cancer types (21). While ID1 is not a classical oncogene, it promotes tumor progression by sustaining a progenitor-like state characterized by enhanced proliferative capacity, resistance to apoptosis and maintenance of stemness properties. Clinical studies have consistently demonstrated that elevated ID1 expression correlates with advanced disease stage, metastatic progression, and poor patient outcomes in diverse malignancies (22–25). In pancreatic adenocarcinoma, emerging evidence suggests that ID1 contributes to tumor aggressiveness by promoting epithelial-mesenchymal transition (EMT) and conferring resistance to chemotherapy (26–29). However, the regulatory mechanisms controlling ID1 expression in PAAD, particularly under the hypoxic condition characteristic of the PAAD microenvironment, remain poorly understood. This knowledge gap is particularly significant given that hypoxia is known to modulate the expression of numerous transcription factors and oncogenic regulators through both HIF-dependent and independent pathways. The potential intersection between hypoxic signaling and ID1-mediated oncogenic processes in PAAD represents an important area of investigation with potential therapeutic implications. Therefore, this study aims to elucidate how hypoxia regulates ID1 expression in pancreatic adenocarcinoma (PAAD), with a specific focus on post-translational modifications mediated by the E3 ubiquitin ligase TRIM21. In this study, we uncover a novel hypoxia-mediated regulatory mechanism driving PAAD progression through ID1 stabilization. Our findings demonstrate that ID1 is not only highly expressed in PAAD but is further induced under hypoxic conditions (26, 29, 30), correlating with enhanced tumor aggressiveness. Besides, hypoxia promotes ID1 protein stability through transcriptional suppression of TRIM21, the E3 ubiquitin ligase responsible for ID1 degradation. Collectively, these findings provide the first evidence of post-translational regulation of ID1 by microenvironmental hypoxia via PAAD, offering new insights into tumor adaptation to low oxygen conditions. The identification of this regulatory axis reveals TRIM21-mediated ID1 degradation as a potential therapeutic vulnerability in PAAD. Targeting this pathway may represent a promising strategy to counteract hypoxia-induced metastatic potential in this lethal malignancy.

## 2 Materials and methods

### 2.1 Cell culture

The human pancreatic cancer cell lines (PANC-1, SW1990, and AsPC-1) were obtained from the Cell Bank of the Chinese Science Academy (Shanghai, China). Human pancreatic duct epithelial cell (HPDEC) was obtained from American Type Culture Collection (ATCC). PANC-1 and HPDEC were maintained in Dulbecco’s modified Eagle’s medium (DMEM) supplemented with 10% fetal bovine serum (HyClone). AsPC-1 and SW1990 cells were maintained in RPMI 1640 medium supplemented with 10% fetal bovine serum (HyClone). All the cell lines were checked routinely for mycoplasma and tested cytogenetically to ensure their authentication.

### 2.2 Reagents and antibodies

The primary antibodies used in western blot, immunohistochemistry and confocal assays included antibodies against ID1 (#67827, proteintech), DYKDDDDK Tag (#14793, Cell Signaling Technology), Ubiquitin (#20326, Cell Signaling Technology), β-Tubulin ((#10094, proteintech), TRIM21 (#12108, proteintech), GAPDH (#60004, proteintech) and β-ACTIN (# 66009-1, Proteintech). MG132 (HY‐13259), Chloroquine (HY-17589A) and Cycloheximide (HY‐12320) were purchased from MCE (Shanghai).

### 2.3 DNA constructs

Polymerase chain reaction (PCR)-amplified human ID1 and TRIM21 were cloned into pLVX-Puro-V5 and pLVX-IRES-Neo-3xFlag vector. Ubiquitin was cloned into the pCDNA 3.0-HA vector(HA-Ub). shRNA was constructed via ligation of an oligonucleotide targeting human ID1 and TRIM21 into an AgeI/Ecor1-digested pLKO.1 vector. The shRNA target sequences are listed in **Table 1**.

**Table 1:** Sequence of shRNA target primers.

shRNA	Sequence
shID1-1	CCTACTAGTCACCAGAGACTT
shID1-2	GCGAGGTGGTACTTGGTCTGT
shTRIM21-1	GAGTTGGCTGAGAAGTTGGAA
shTRIM21-2	TGGCATGGTCTCCTTCTACAA

### 2.4 Transwell assay

For the transwell assay, pancreatic cancer cells were seeded in the upper chamber with serum-free medium. A total of 1 × 10^6^ cells were used for the migration assay, while 1.5 × 10^6^ cells were used for the invasion assay. The chamber was either coated or left uncoated with Matrigel (Corning) to assess cell invasion and migration. The lower chamber was filled with 500 μL of medium containing 10% FBS. After 48 hours of incubation, penetrating cells were fixed with 4% methanol for 15 minutes and then stained with 0.1% crystal violet solution for 15 minutes. The Transwell chambers were washed with PBS, and non-invading cells were gently removed using a cotton swab. Membranes were then randomly imaged under an optical microscope.

### 2.5 Proliferation assays

For the Cell Counting Kit-8 (CCK8) assay, 5000 cells were seeded in 96-well plates, and 10 μL of CCK8 solution was added to each well, followed by a 90-minute incubation. Cell viability was assessed by measuring absorbance at 450 nm. For the colony formation assay, cells were seeded at a density of 1000 cells per well and incubated for 2 weeks. Afterward, cells were washed with phosphate-buffered saline (PBS), fixed with 4% paraformaldehyde, and stained with 0.1% crystal violet. Following thorough washing to remove excess dye, colonies were photographed and counted, with each colony containing at least 50 cells.

### 2.6 Reverse-transcription and quantitative RT-PCR

Total RNA was extracted using TRIzol reagent (TaKaRa) and reverse transcribed with Maxima Reverse Transcriptase (Yeasen) following the manufacturer’s instructions. cDNAs were quantified by quantitative RT-PCR using SYBR Green dye (Yeasen). The results were normalized to β-ACTIN mRNA levels. The RT-PCR primer sequences are listed in **Table 2**.

**Table 2:** Sequence of primers used in RT-qPCR.

Primer	Sequence
Human ID1-F	CTGCTCTACGACATGAACGG
Human ID1-R	GAAGGTCCCTGATGTAGTCGAT
Human β-ACTIN -F	CACTCTTCCAGCCTTCCTTC
Human β-ACTIN -R	GTACAGGTCTTTGCGGATGT

### 2.7 Immunofluorescence

Cells were seeded in confocal dishes and allowed to fully adhere. After complete attachment, they were subjected to hypoxic treatment for 12 hours. The cells were then fixed with 4% paraformaldehyde for 15 minutes, followed by permeabilization with 0.1% Triton X-100. After blocking with 5% BSA blocking buffer, the cells were incubated with the primary antibody overnight at 4°C, followed by incubation with the secondary antibody. Nuclei were visualized using DAPI staining. Finally, images were captured using a Leica confocal microscope.

### 2.8 Western blot

Western blotting was performed using the cell lysate according to standard protocols. Briefly, proteins were separated by SDS‐PAGE, transferred to nitrocellulose filter membranes, and subjected to immunoblotting with antibodies. All antibodies were validated by the commercial vendor. Densitometric analyses of protein bands were performed using ImageJ software. Nuclear and cytoplasmic protein was prepared by Nuclear and Cytoplasmic Protein Extraction Kit (Beyotime, P0028) according to the manufacturer’s instructions.

### 2.9 Transfection

To obtain stable cell lines, the lentiviral expression vector and packaging vectors (pMD2. G and psPAX) were transfected into HEK293T‐cells to produce the virus. After collecting the viral supernatant fractions at 48 and 72 h, target cells were infected and selected with puromycin or G418. Cells were plated at a density of 5 × 10^6^ cells per 10-mm dish or 1 × 10^5^ cells per well in a six-well plate 18 h before transfection. Cells were transfected with various plasmids, various packed virus and shRNA using lipofectamine 2000 (Invitrogen) according to the vendor’s instructions.

### 2.10 Mass spectrometry analyses of proteins

ID1-associated proteins were analyzed by LC–MS/MS. Briefly, Flag-EZH2-associated proteins from the immunoprecipitation assay were acetone-precipitated overnight at −20°C and resuspended in 50 mM ammonium bicarbonate buffer containing RapiGest (Waters Corp). The sample was heated to 95°C for ten min and then allowed to cool, and 100 ng of sequencing-grade modified trypsin (Promega) was added. The digestion proceeded at 37°C overnight and was analyzed with a hybrid Q-Exactive mass spectrometer (Thermo Fisher Scientific). Proteins were analyzed by comparing the fragment spectra against those in the SwissProt protein database. All peptide matches were initially filtered based on mass measurement errors and Xcorr and Corr scores and further manually validated for peptide identification.

### 2.11 Mice

Six-week-old male nude mice (n = 5 per group) were subcutaneously injected with 3 × 10^6^ gene-modified PANC-1 cells in 100 μL of PBS into the left axilla. Tumor volume was calculated using the formula V = ab²/2, where a is the base diameter and b is the perpendicular diameter. All mice were housed in specific-pathogen-free conditions, and the study was approved by the Institutional Animal Care and Use Committee of Peking Third Hospital. The animals were fed a standard rodent diet, and all procedures complied with relevant ethical guidelines for animal research.

### 2.12 Immunohistochemistry

The slides (188105w, CITOGLAS) were treated with 1× Tris-EDTA antigen retrieval buffer (C1038, Solarbio) and incubated with endogenous peroxidase blocker (3% hydrogen peroxide, GK600505, Genetech). After blocking with 5% BSA, the slides were incubated with the primary antibody (ID1, Ki-67 and TRIM21) overnight at 4°C, followed by incubation with the secondary antibody. The slides were then developed using DAB color development solution and counterstained with hematoxylin.

### 2.13 Bioinformatic analysis

ID1expression data and clinical information for pancreatic adenocarcinoma (PAAD) were obtained from The Cancer Genome Atlas (TCGA) database. ID1 expression levels in tumor and normal tissues were analyzed using the UALCAN database (http://ualcan.path.uab.edu), which provides user-friendly access to TCGA RNA-seq and clinical data. The association between ID1 expression and patient prognosis was evaluated through Kaplan-Meier survival analysis using the Kaplan-Meier Plotter database (http://kmplot.com), which integrates gene expression and survival data from multiple sources including TCGA.

### 2.14 Cycloheximide chase assay

Cells were seeded in 6-well plates and cultured to approximately 50% confluence. Cycloheximide (CHX; MCE, HY‐12320) was added to the culture medium at a final concentration of 20μM to inhibit protein synthesis. Cells were harvested at indicated time points (0, 4, 8, 12 hours) after CHX treatment. Total protein was extracted using RIPA buffer supplemented with protease inhibitors and subjected to western blotting to analyze the degradation rate of the target protein.

### 2.15 Co-immunoprecipitation

Cells were transfected with plasmids encoding Flag-tagged and/or proteins using Lipofectamine 2000 according to the manufacturer’s protocol. 36 hours post-transfection, cells were exposed to hypoxic conditions (3% O_2_) for an additional 12 hours. Cells were then washed with cold PBS and lysed in NP-40 lysis buffer (50 mM Tris-HCl pH 7.5, 150 mM NaCl, 1% NP-40) supplemented with protease and phosphatase inhibitors. Lysates were cleared by centrifugation at 12,000 × g for 10 minutes at 4°C. A small portion of the lysate was retained as input. The remaining lysate was incubated with anti-FLAG M2 magnetic beads (Sigma-Aldrich, Cat# M8823) overnight at 4°C with gentle rotation. Beads were washed three times with lysis buffer, and bound proteins were eluted by boiling in SDS sample buffer. Input and IP: Flag samples were subjected to SDS-PAGE and western blotting using appropriate antibodies to analyze protein expression and interactions under hypoxic and normoxia conditions.

### 2.16 Quantification and statistical analysis

All data represent the mean ± SD of three independent experiments/samples unless specifically indicated. The statistical analysis was conducted using the two-tailed unpaired Student’s t test. GraphPad was used for statistical analysis. P value of 0.05 was considered a borderline for statistical significance.

## 3 Results

### 3.1 ID1 overexpression promotes tumor aggressiveness and predicts poor outcomes in PAAD

**Figure 1:**
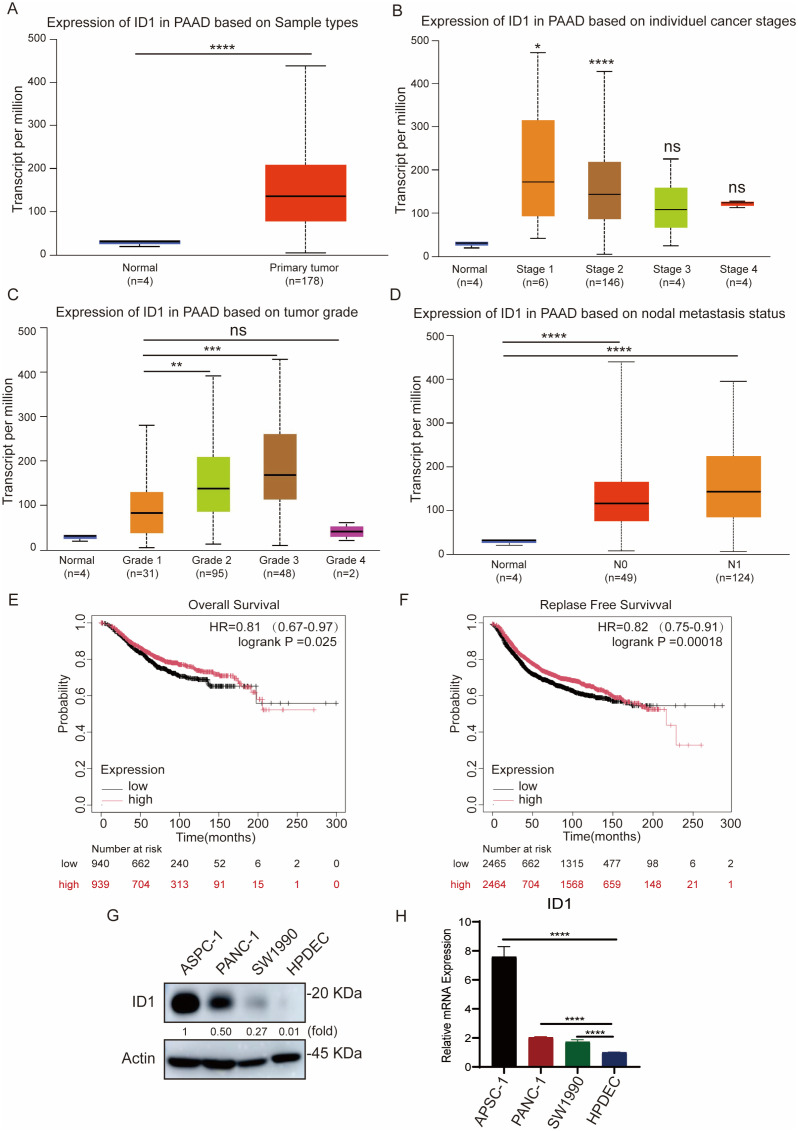
ID1 overexpression promotes tumor aggressiveness and predicts poor outcomes in PAAD. **(A)** ID1 expression levels in normal and PAAD tissues based on UALCAN database analysis. **(B)** ID1 expression stratified by cancer stage in PAAD, showing significantly elevated levels in stage I and II tumors. **(C)** ID1 expression levels in PAAD stratified by tumor grade, with progressive increases observed from grade 1 to grade 3. **(D)** ID1 expression based on nodal metastasis status in PAAD patients. **(E)** Kaplan–Meier survival analysis of overall survival (OS) in PAAD patients with high versus low ID1 expression. **(F)** Kaplan–Meier survival analysis of relapse-free survival (RFS) in PAAD patients with high versus low ID1 expression. **(G)** Western blot analysis of ID1 protein expression in PAAD cell lines (AsPC-1, PANC-1, SW1990) and normal pancreatic duct epithelial cells (HPDEC). **(H)** Quantitative RT-PCR analysis of ID1 mRNA expression in PAAD cell lines (AsPC-1, PANC-1, SW1990) and normal pancreatic duct epithelial cells (HPDEC). *Statistical significance: *P < 0.05; **P < 0.01; ***P < 0.001; ****P < 0.0001; ns, not significant.

To investigate the clinical significance of ID1 inPAAD, we analyzed ID1 expression by the UALCAN database. ID1 expression was significantly upregulated in PAAD tissues compared to normal pancreatic tissues (**Figure 1A**). Further analysis revealed that ID1 expression was markedly elevated in tumors at stage I and stage II (**Figure 1B**), and increased progressively with tumor grade from grade 1 to grade 3 (**Figure 1C**), suggesting a correlation with disease advancement. Although ID1 levels appeared higher in grade 4 tumors, the difference was not statistically significant, possibly due to the limited sample size in this group. In addition, ID1 expression was significantly higher in metastatic tumors at both N0 and N1 stages (**Figure 1D**), indicating a potential role in tumor metastasis. Kaplan-Meier survival analysis using the Kaplan-Meier Plotter database demonstrated that high ID1 expression was strongly associated with poorer overall survival (OS), disease-free survival (DFS), and disease-free metastasis survival (DFMS) in PAAD patients (**Figures 1E, F**, **Supplementary Figure S1A**).

Consistent with tissue analysis, elevated ID1 expression was also observed in multiple human pancreatic cancer cell lines, including AsPC-1, PANC-1, and SW1990, compared with normal human pancreatic duct epithelial cells (HPDEC) (**Figures 1G, H**).

Collectively, these findings indicate that ID1 is highly expressed in PAAD and is positively associated with the clinical aggressiveness of the disease.

### 3.2 Hypoxia potentiates ID1-driven malignant progression in PAAD through enhanced proliferation and metastatic capacity

**Figure 2:**
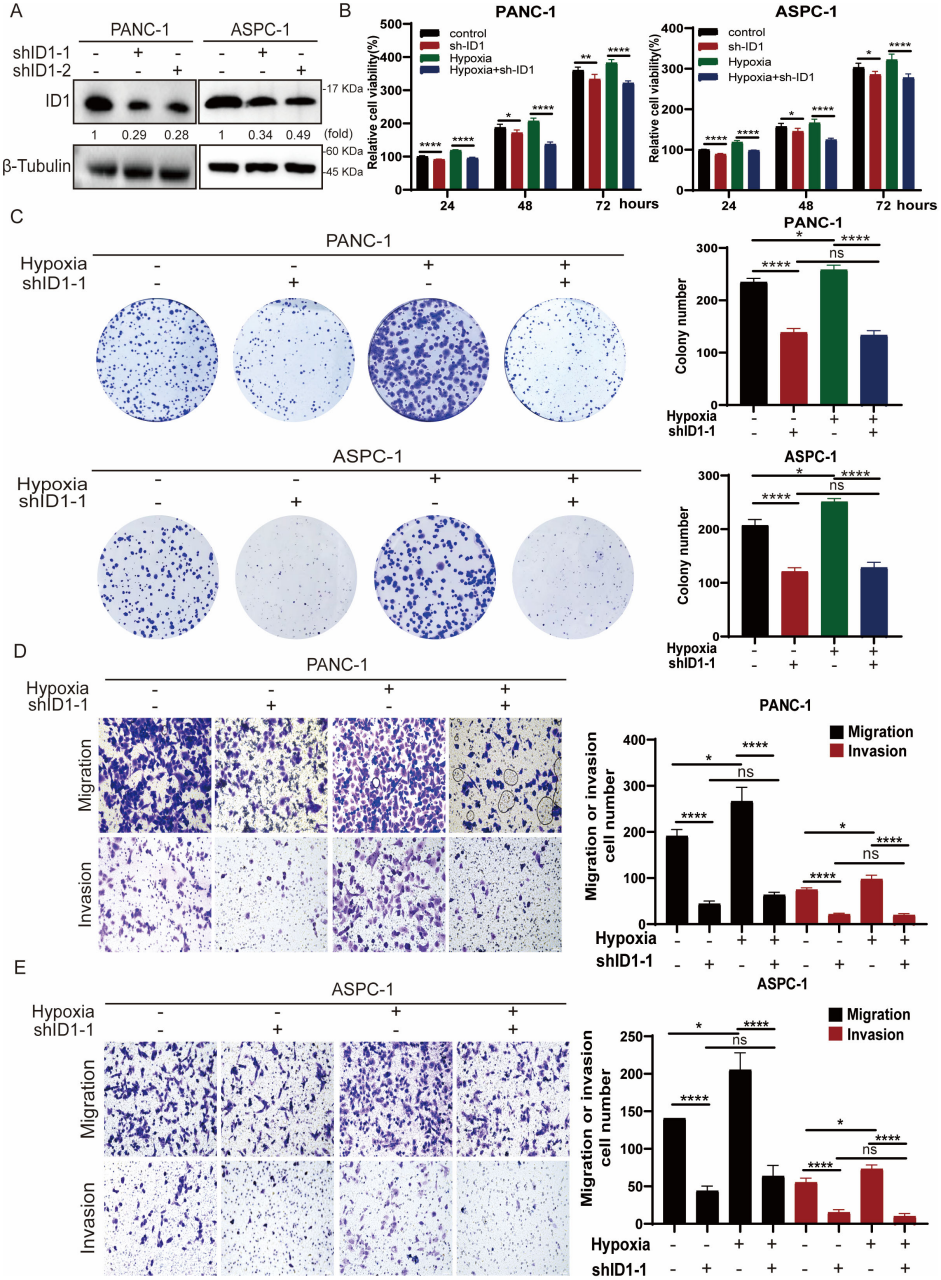
Hypoxia potentiates ID1-driven malignant progression in PAAD through enhanced proliferation and metastatic capacity. **(A)** ID1 was silenced in the indicated cell lines using ID1-targeting shRNA. **(B)** The indicated cells were cultured under normoxic (21% O_2_) or hypoxic (3% O_2_) conditions for the indicated durations. Cell proliferation was measured using the CCK-8 assay. “Relative cell viability (%)” represents viability normalized to the corresponding normoxic control at 24 h Data are presented as mean ± SD from five independent experiments (n = 5). **(C)** Colony formation assays were performed with PANC-1 and AsPC-1 cells cultured under normoxic or hypoxic conditions for two weeks. Colonies were stained and counted. Data are presented as mean ± SD from three independent experiments (n = 3). **(D, E)** Cell migration and invasion of PANC-1 and ASPC-1were assessed by Transwell assays under normoxic or hypoxic conditions. Representative images of migrated/invaded cells stained on the membrane are shown. Scale bar, 50 μm. *Statistical significance: *P < 0.05; **P < 0.01; ****P < 0.0001; ns, not significant.

Given that hypoxia is a hallmark of the tumor microenvironment in pancreatic adenocarcinoma, we simulated this condition *in vitro* by culturing cells under hypoxic conditions to better understand its influence on tumor cell behavior. To explore the functional role of ID1 in this context, we established stable ID1 knockdown cell lines (**Figure 2A**). A series of functional assays were then conducted to evaluate the effects of ID1 silencing on cell proliferation, migration, and invasion. Both CCK-8 and colony formation assays demonstrated that ID1 promotes the proliferation of PANC-1 and AsPC-1 cells under normoxic conditions. Notably, hypoxia further enhanced the pro-proliferative effects of ID1 in pancreatic cancer cells (**Figures 2B, C**). Transwell assays confirmed that ID1 knockdown significantly impaired the migratory and invasive capacities of PANC-1 and AsPC-1 cells (**Figures 2D, E**). Moreover, hypoxic conditions amplified the ability of ID1 to promote migration and invasion, underscoring its critical role in PAAD progression under hypoxic stress.

### 3.3 Hypoxia stabilizes ID1 protein by inhibiting ubiquitin-mediated degradation in pancreatic cancer cells

**Figure 3:**
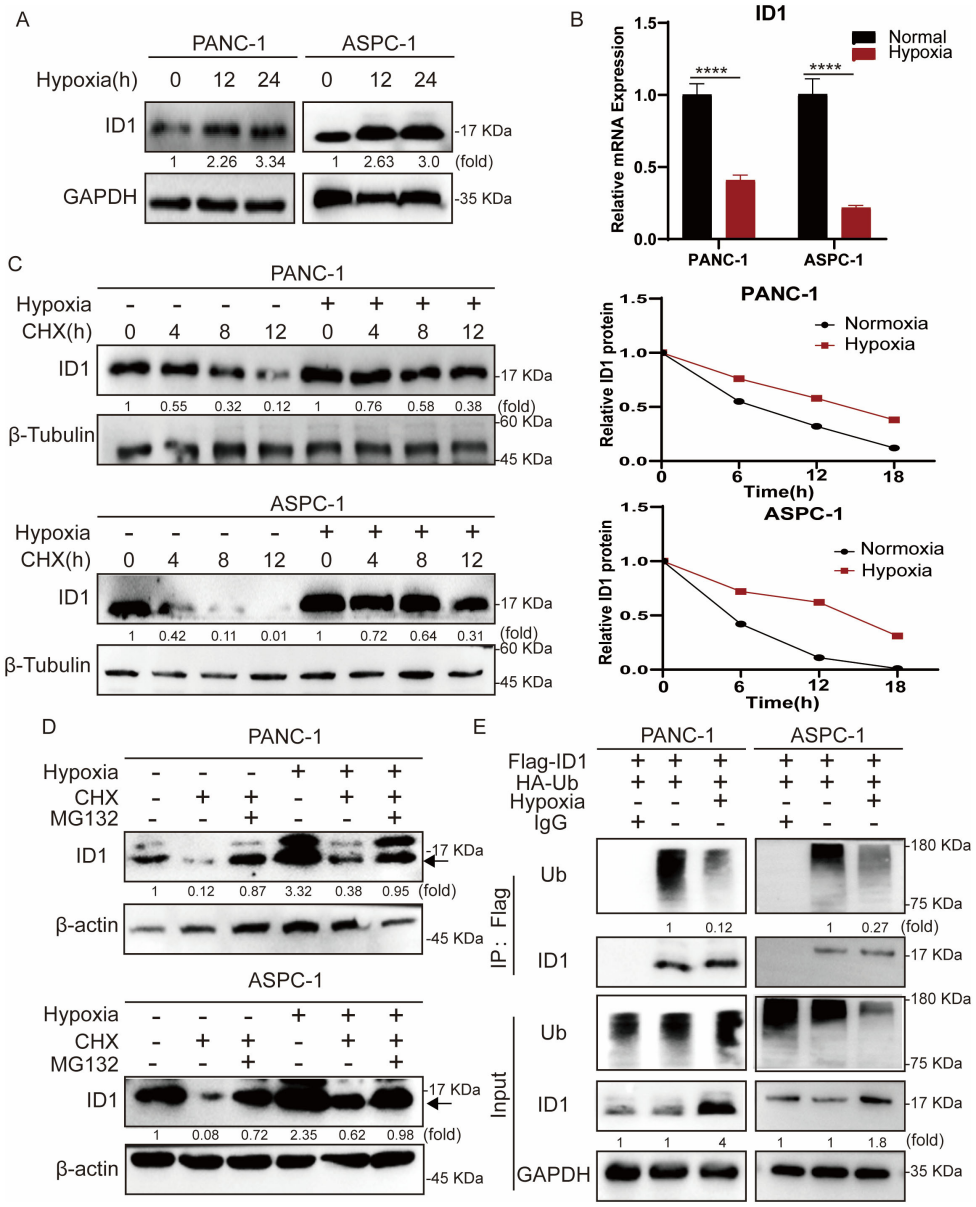
Hypoxia stabilizes ID1 protein by inhibiting ubiquitin-mediated degradation in pancreatic cancer cells. **(A)** Immunoblot analysis of ID1 protein expression under normoxic (21% O_2_) and hypoxic (3% O_2_) conditions. **(B)** ID1 mRNA levels were measured by quantitative RT-PCR after 24-hour exposure to hypoxic (3% O_2_) or normoxic (21% O_2_) conditions. (n = 9). **(C)** Cycloheximide (CHX) chase assay (20 μg/mL) was conducted to evaluate ID1 protein stability. Cells were treated with CHX to block protein synthesis for 0, 6, 12, 18 hours after cells subjected to hypoxia for 12 hours, and ID1 levels were assessed at indicated time points to determine the degradation rate. **(D)** Immunoblot analysis of ID1 protein was performed following treatment with the proteasome inhibitor MG132 (20 μM) and CHX (20 μM) for 18 hours, after cells were subjected to hypoxia for 12 hours under normoxic and hypoxic conditions. **(E)** Ubiquitination assay was conducted by immunoprecipitating Flag-tagged ID1 using anti-Flag magnetic beads, followed by immunoblotting with anti-ubiquitin antibodies. The results show that hypoxia markedly reduces ID1 polyubiquitination, indicating that hypoxic conditions suppress its ubiquitin-mediated proteasomal degradation. ****P < 0.0001.

To further investigate the mechanism by which ID1 promotes pancreatic cancer growth under hypoxic conditions, we first examined ID1 protein expression following hypoxia treatment (**Figure 3A**). Our results showed that ID1 protein levels were significantly elevated under hypoxia. As protein regulation can occur at both the transcriptional and post-translational levels (31), we next measured ID1 mRNA expression (**Figure 3B**, **Supplementary Figures S3A, B**). Interestingly, ID1 mRNA levels were decreased under hypoxic conditions, suggesting that the increased protein expression is not driven by transcriptional upregulation.

To further validate this observation, we treated cells with the protein synthesis inhibitor cycloheximide (CHX). Compared to hypoxic conditions, ID1 protein levels declined more rapidly under normoxia following CHX treatment, indicating enhanced post-translational degradation of ID1 under normoxia (**Figure 3C**). Protein degradation in cells is primarily mediated by either the ubiquitin-proteasome system or the autophagy-lysosome pathway (32, 33). To explore the mechanism regulating ID1 protein stability, we treated cells with the proteasome inhibitor MG132 and autophagy inhibitors CQ (**Figure 3D**, **Supplementary Figure S4A**). MG132 treatment significantly increased ID1 protein levels under normoxia, suggesting that ID1 is degraded via the ubiquitin-proteasome pathway.

Finally, we assessed the ubiquitination of ID1 in pancreatic cancer cells (**Figure 3E**). Ubiquitination of ID1 was readily detected under normoxic conditions but was markedly reduced following hypoxia exposure. Together, these data establish that hypoxia stabilizes ID1 by specifically impairing its ubiquitin-proteasomal degradation, revealing a post-translational mechanism for ID1 accumulation in pancreatic cancer.

### 3.4 Hypoxia-mediated TRIM21 silencing orchestrates ID1 stabilization to promote pancreatic tumorigenesis

**Figure 4:**
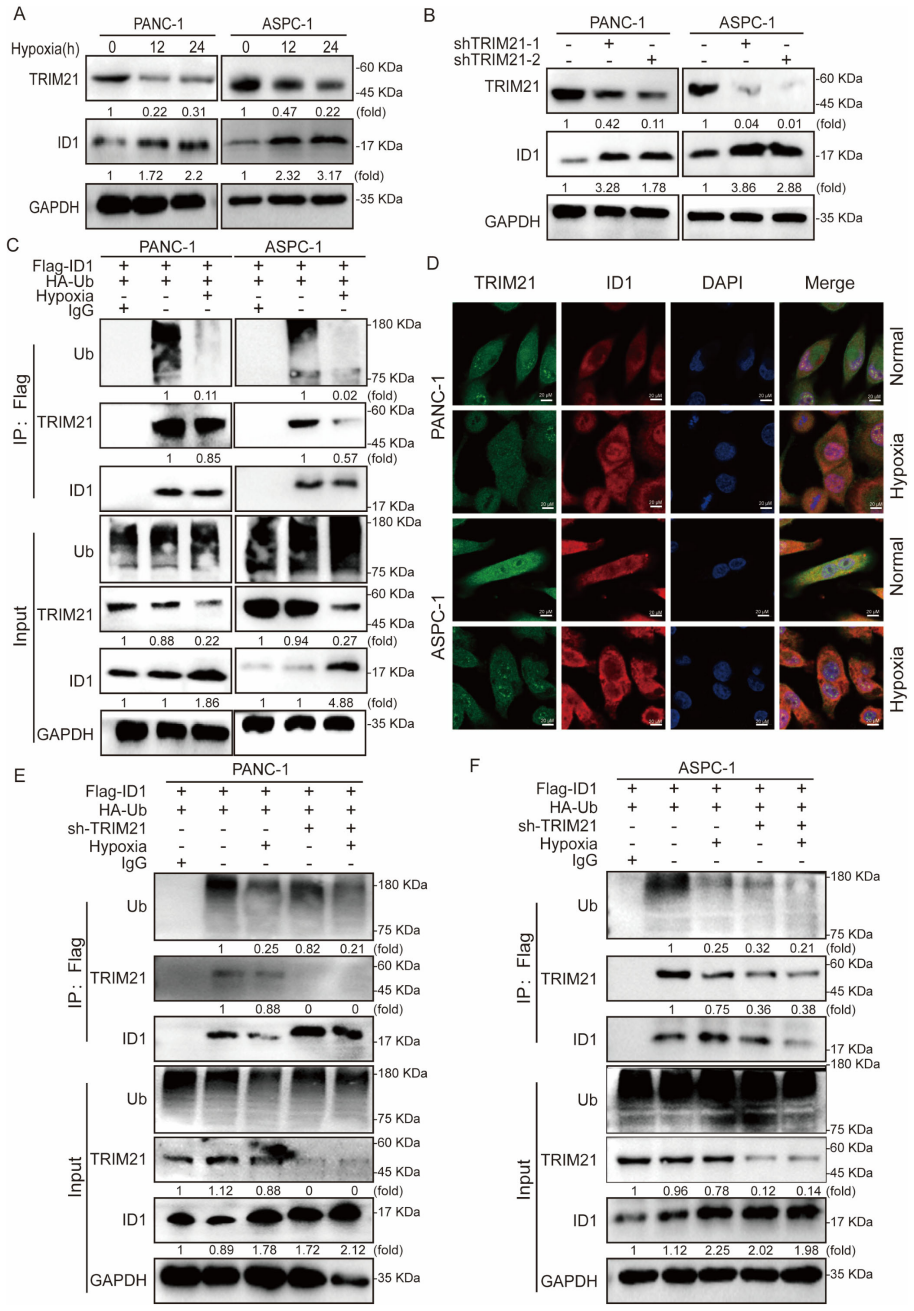
Hypoxia-mediated TRIM21 silencing orchestrates ID1 stabilization to promote pancreatic tumorigenesis. **(A)** Immunoblot analysis of TRIM21 expression under hypoxic (3% O_2_) versus normoxic (21% O_2_) conditions. **(B)** Immunoblotting of ID1 protein levels in control and TRIM21-knockdown cells under normoxic and hypoxic conditions. **(C)** Co-immunoprecipitation (Co-IP) assay demonstrating the interaction between ID1 and TRIM21 under normoxia and hypoxia. Cell lysates from PANC-1 and AsPC-1 cells were immunoprecipitated using anti-Flag antibody to pull down Flag-tagged ID1. “Input” lanes show whole-cell lysates before immunoprecipitation; “IP: Flag” lanes indicate proteins co-precipitated with Flag-ID1. Lane 1 represents the negative control (IgG control IP), lane 2 corresponds to the normoxia-treated group, and lane 3 represents the hypoxia-treated group. **(D)** Immunofluorescence staining showing co-localization (yellow) of ID1 (red) and TRIM21 (green) in cells cultured under normoxia. Hypoxia reduced the degree of co-localization. Nuclei were counterstained with DAPI (blue). Scale bar: 20 μm. **(E, F)** Ubiquitination assays showing polyubiquitinated ID1 levels in control and TRIM21-knockdown cells under normoxic and hypoxic conditions. TRIM21 depletion decreased ID1 ubiquitination, particularly under normoxia.

To elucidate the mechanism underlying ID1 ubiquitination, we conducted mass spectrometry analysis in ID1-overexpressing cells, which identified TRIM21 as a binding partner of ID1 under normoxic conditions. To validate this interaction, we first examined TRIM21 expression under normoxia and hypoxia conditions of PANC-1 and ASPC-1 cells, revealing significant downregulation of TRIM21 under hypoxic conditions (**Figure 4A**). TRIM21 (Tripartite motif-containing protein 21), a member of the TRIM protein family, functions as an E3 ubiquitin ligase that regulates substrate protein ubiquitination and degradation, thereby participating in various cellular processes including innate immunity, antiviral defense, and tumorigenesis. Recent studies have revealed that TRIM21 acts as a tumor suppressor in multiple cancers by targeting and degrading oncoproteins to modulate tumor progression (34, 35).

To determine whether TRIM21 regulates ID1 stability, we generated stable TRIM21-knockdown cell lines and observed a marked restoration of ID1 expression upon TRIM21 depletion (**Figure 4B**). Subsequent co-immunoprecipitation (Co-IP) assays confirmed a direct interaction between ID1 and TRIM21, which was substantially weakened under hypoxia (**Figure 4C**). Furthermore, immunofluorescence analysis demonstrated spatial co-localization of ID1 and TRIM21 under normoxia, whereas hypoxia-induced TRIM21 downregulation led to reduced interaction and concomitant ID1 upregulation (**Figure 4D**, **Supplementary Figures S5A–E**).

Finally, ubiquitination assays in TRIM21-knockdown cells revealed that TRIM21 ablation significantly attenuated ID1 ubiquitination, supporting its role as a critical E3 ligase for ID1 degradation in pancreatic cancer cells (**Figures 4E, F**).

In summary, under hypoxic conditions, ID1 ubiquitination was significantly reduced. Mechanistically, we identified TRIM21 as the E3 ubiquitin ligase responsible for mediating ID1 ubiquitination. Hypoxia primarily caused a marked downregulation of TRIM21 expression, which appears to be the major contributor to the observed decrease in ID1 ubiquitination. Although a modest reduction in the TRIM21–ID1 interaction was also observed under hypoxia, the loss of TRIM21 expression plays a predominant role in this regulation. It is plausible that hypoxia-driven suppression of TRIM21 impairs the recruitment of the ubiquitination machinery, thereby prolonging ID1 protein half-life and enhancing its downstream signaling. Together, these findings suggest that hypoxia promotes ID1 stabilization predominantly through TRIM21 downregulation, thereby enhancing ID1-mediated signaling during tumor progression.

### 3.5 TRIM21 suppresses ID1-mediated tumorigenesis and counteracts hypoxia-induced tumor aggressiveness

**Figure 5:**
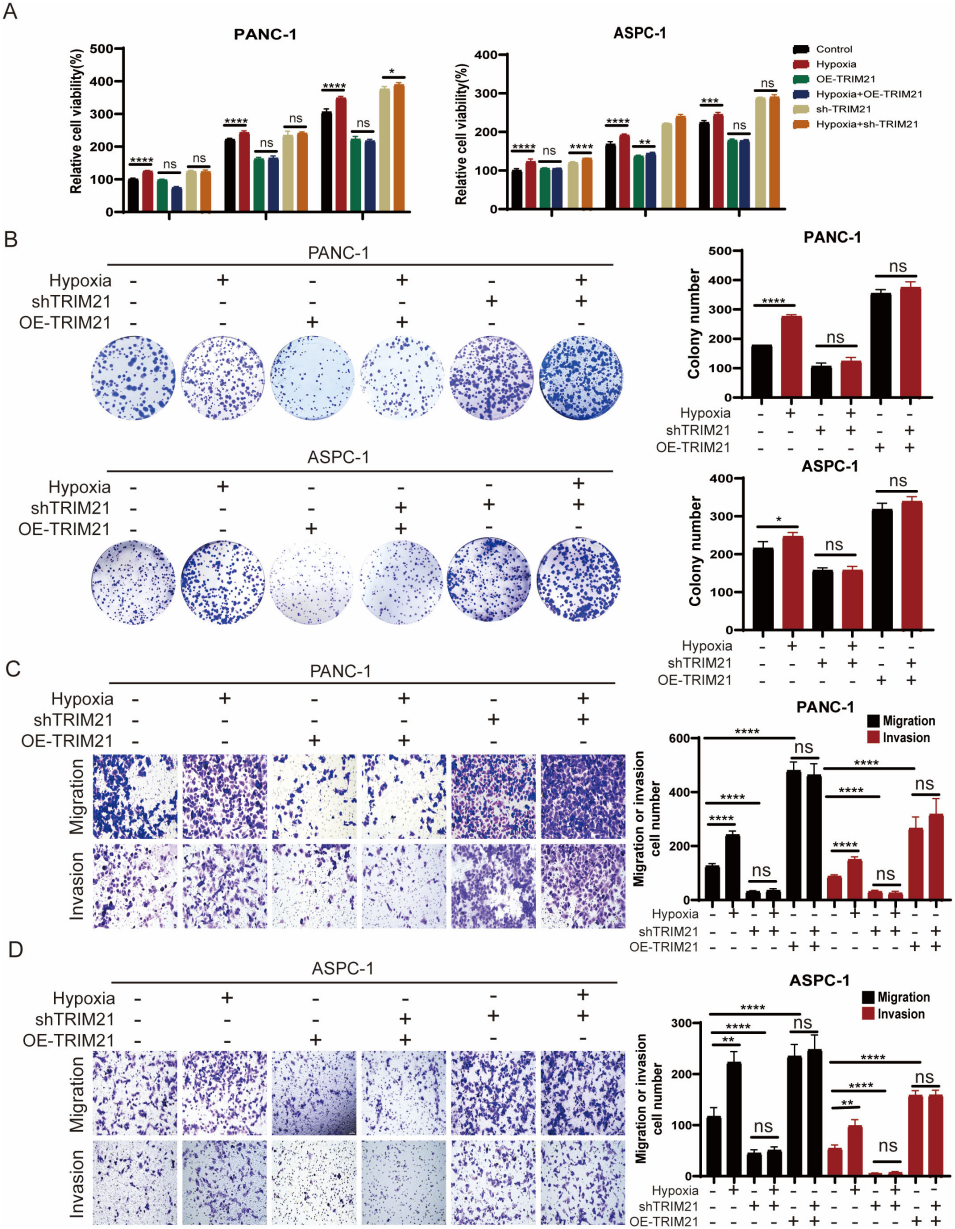
TRIM21 knockdown reverses hypoxia-driven oncogenic phenotypes in PDAC **(A)** PANC-1 and AsPC-1 cells with TRIM21 knockdown, TRIM21 overexpression, or corresponding controls were cultured under normoxic (21% O_2_) or hypoxic (3% O_2_) conditions for 24, 48, and 72 hours. Cell proliferation was assessed using the Cell Counting Kit-8 (CCK-8) assay. “Relative cell viability (%)” represents viability normalized to the corresponding normoxic control at 24 h Data are presented as mean ± SD from five independent experiments (n = 5). **(B)** Colony formation assays were performed in PANC-1 and AsPC-1 cells with TRIM21 knockdown, TRIM21 overexpression, or corresponding controls under normoxic or hypoxic conditions for two weeks. Colony numbers were quantified. Data are shown as mean ± SD from three independent experiments (n = 3). **(C, D)** Transwell migration and invasion assays were conducted in PANC-1 and AsPC-1 cells under normoxia or hypoxia, with TRIM21 knockdown, TRIM21 overexpression, or control treatments. Representative images of crystal violet–stained cells are shown. Scale bars, 50 μm. Data are presented as mean ± SD. *Statistical significance: *P < 0.05; **P < 0.01; ***P < 0.001; ****P < 0.0001; ns, not significant.

Our findings demonstrate that hypoxia stabilizes ID1 protein by downregulating TRIM21 expression. To investigate whether TRIM21 directly modulates ID1-driven tumorigenesis, we performed functional characterization using stable TRIM21 knockdown and overexpression cell lines. Compared to control cells, both CCK-8 and colony formation assays showed that TRIM21 overexpression significantly inhibited the proliferation of PANC-1 and AsPC-1 cells under both normoxic and hypoxic conditions (**Figures 5A, B**, **Supplementary Figure S6A**). Similarly, Transwell assays revealed that TRIM21 overexpression markedly suppressed cell migration and invasion (**Figures 5C, D**). Notably, there were no significant differences between normoxic and hypoxic conditions in TRIM21-overexpressing cells, suggesting that elevated TRIM21 levels effectively counteract hypoxia-induced ID1 stabilization. These results parallel the phenotypes observed in ID1 knockdown models, further supporting the role of TRIM21 as a suppressor of ID1-driven tumor progression.

Conversely, TRIM21 knockdown enhanced cell proliferation, migration, and invasion under both normoxic and hypoxic conditions (**Figures 5A–D**). In TRIM21-deficient cells, the phenotypic differences between normoxia and hypoxia were attenuated, likely because TRIM21 expression was already suppressed, limiting further ID1 accumulation under hypoxia. This indicates that the oncogenic effects of hypoxia are at least partially mediated by TRIM21 downregulation, and underscores TRIM21 as a key modulator of hypoxia-adaptive tumor behavior via post-translational control of ID1.

### 3.6 The TRIM21-ID1 regulatory axis controls pancreatic tumor growth through ubiquitin-mediated proteostasis

**Figure 6:**
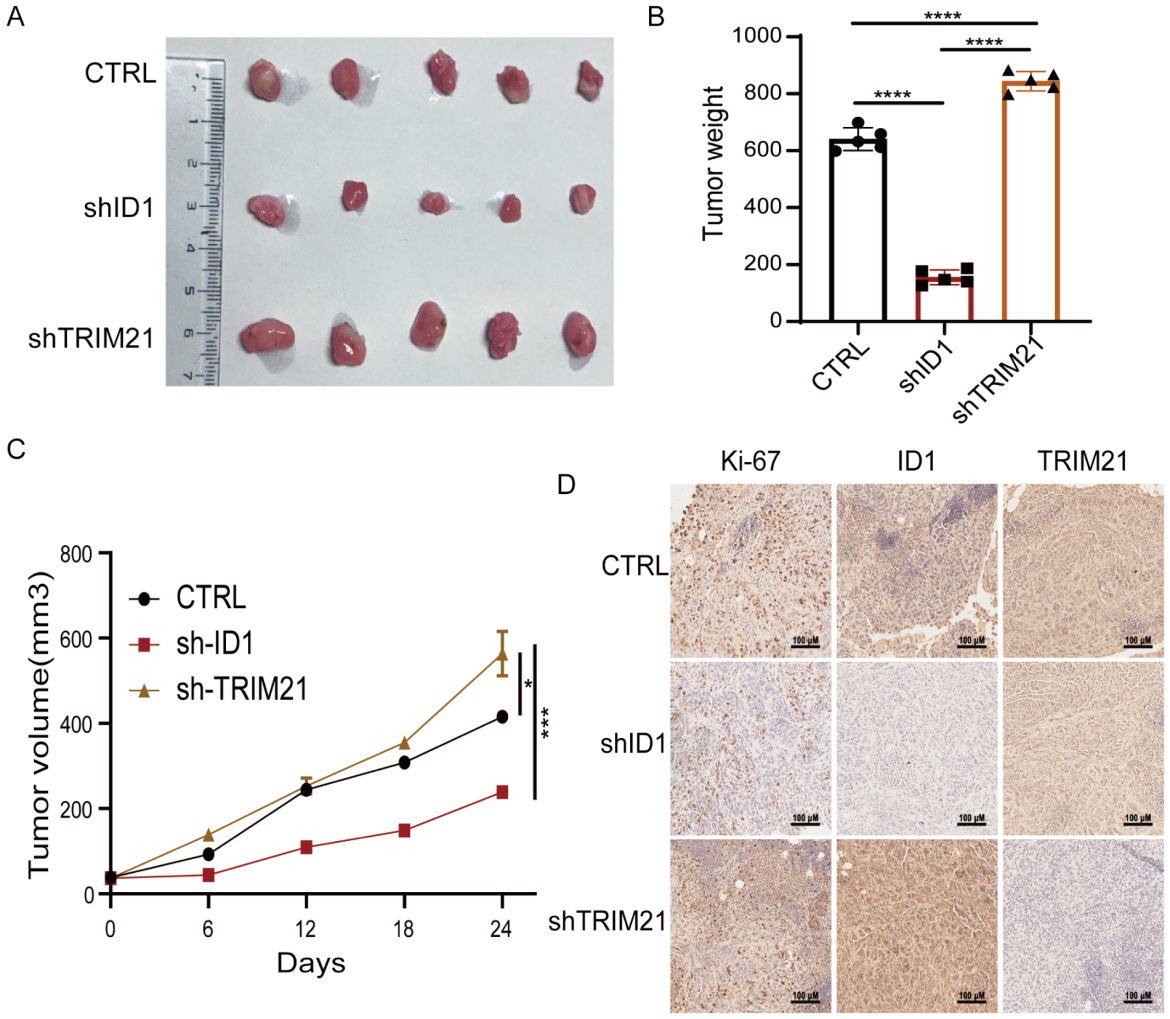
The TRIM21-ID1 regulatory axis controls pancreatic tumor growth through ubiquitin-mediated proteostasis. **(A–D)** PANC-1 cells with stable knockdown of ID1 and TRIM21 were implanted into athymic nude mice via subcutaneous injection. Tumor growth was examined 21 days after injection. Representative tumor xenografts were shown (*n* = 5 mice per group) **(A)**. Tumor weights were calculated **(B)**. Tumor growth was measured every other day beginning on day 21, and tumor volumes were calculated **(C)**. Immunohistochemical analyses of tumor sections with the indicated antibodies were performed. Representative images are shown **(D)**. Scale bar, 100 μm. *Statistical significance: *P < 0.05; ***P < 0.001; ****P < 0.0001.

To further investigate the roles of ID1 and TRIM21 in PAAD proliferation, we performed subcutaneous xenograft experiments using PANC-1 cells with stable knockdown of either ID1 or TRIM21 in nude mice. We found that knockdown of ID1 significantly suppressed tumor growth, whereas silencing TRIM21 led to accelerated tumor progression (**Figures 6A–C**). Immunohistochemical analysis revealed that Ki-67 expression was markedly decreased in ID1-deficient tumors but increased in TRIM21-deficient tumors. Notably, tumors with TRIM21 knockdown also exhibited elevated ID1 expression (**Figure 6D**). These findings suggest that TRIM21 promotes pancreatic tumor growth *in vivo*, at least in part, by regulating the expression of ID1.

## 4 Discussion

Pancreatic adenocarcinoma is characterized by a profoundly hypoxic tumor microenvironment, a hallmark feature that drives tumor aggressiveness and confers resistance to conventional therapies (36, 37). Beyond its well-documented roles in promoting angiogenesis and metabolic reprogramming, hypoxia activates a cascade of adaptive signaling pathways that collectively enhance tumor cell survival, proliferation and metastatic dissemination (38, 39). In this study, we elucidate a novel molecular mechanism linking hypoxia to PAAD progression, demonstrating that oxygen deprivation stabilizes the oncogenic protein ID1 by suppressing its E3 ubiquitin ligase, TRIM21. This finding not only provides mechanistic insight into the hypoxic regulation of ID1, but also aligns with clinical observations that hypoxic PAAD tumors exhibit heightened aggressiveness and diminished therapeutic responsiveness. Given the central role of hypoxia in disease progression, therapeutic strategies targeting hypoxia-responsive pathways-particularly the TRIM21-ID1 axis——may present a promising avenue for disrupting microenvironment-driven malignancy. Future studies should explore whether pharmacological inhibition of this axis can sensitize hypoxic tumors to existing therapies, thereby improving clinical outcomes for this malignancy.

ID1 has emerged as a pivotal regulator in PAAD progression, where its overexpression correlates with enhanced proliferation capacity, invasive potential, and stem-like properties——key hallmarks of aggressive disease progression (12, 21, 25–27, 29, 30). Our data provides compelling evidence that hypoxia-mediated stabilization of ID1 exacerbates these tumor-promoting effects, while TRIM21 knockdown mitigates ID1-driven oncogenicity. Notably, ID1 upregulation has been implicated in therapeutic resistance across multiple malignancies, suggesting that its hypoxia-induced stabilization may contribute to the recalcitrance of PAAD to conventional radiotherapy and chemotherapy. The reversible suppression of ID1 upon TRIM21 restoration underscores its dynamic regulation and nominates ID1 as both a promising predictive biomarker and a tractable therapeutic target. Given these findings, further investigation is warranted to determine whether pharmacological inhibition of ID1 can reverse hypoxia-associated treatment resistance, thereby resensitizing PAAD tumors to standard-of-care therapies. Such strategies could potentially disrupt the vicious cycle of hypoxia-driven aggressiveness and improve clinical outcomes for this lethal malignancy.

In addition, our study revealed a significant increase in ID1 mRNA levels following 72 hours of hypoxic exposure, indicating the activation of transcriptional regulatory pathways in response to prolonged oxygen deprivation. These findings support a model in which ID1 expression is dynamically regulated in a time-dependent manner—initially through post-translational stabilization and subsequently via transcriptional activation. This dual-level control highlights a potential mechanistic link between acute cellular stress responses and long-term adaptation within the tumor microenvironment. Furthermore, it underscores the intimate interplay between microenvironmental cues and key oncogenic regulators like ID1, which may collectively drive tumor growth, progression, and therapeutic resistance in pancreatic cancer.

The ubiquitin-proteasome system plays a pivotal role in maintaining oncoprotein homeostasis, with E3 ubiquitin ligases like TRIM21 serving as a critical regulator of protein turnover (40–42). Our work identifies a previously unrecognized mechanism in PAAD pathogenesis, demonstrating that TRIM21-mediated ubiquitination as a key ID1. This finding expands our understanding of post-translational regulation in PAAD and highlights the therapeutic potential of modulating ubiquitination pathways. Of particular translational relevance is the emergence of proteolysis-targeting chimeras (PROTACs) as a ground breaking strategy for targeting traditionally “undruggable” oncoproteins through hijacking the ubiquitin-proteasome system (43, 44). Given our findings, TRIM21-based PROTACs may be developed specifically degrade ID1 in PAAD, especially in hypoxic tumor areas where malignancy is driven by ID1 stability. This approach might get beyond the current restrictions on directly targeting ID1 and offer aggressive PAAD subtypes a precision therapeutic approach.

Our study elucidates a novel mechanistic link between tumor microenvironmental stress and oncogenic signaling through ubiquitin-mediated protein stabilization, establishing the hypoxia TRIM21-ID1 axis as a critical regulator of PAAD pathogenesis. These findings not only provide fundamental insights into the molecular drivers of PAAD progression but also reveal actionable therapeutic vulnerabilities. Specifically, the identified axis presents a promising intervention strategy: developing TRIM21-based therapeutic approaches to restore physiological ID1 degradation. Future investigations should focus on translating these mechanistic discoveries into clinically viable strategies that may overcome the current therapeutic challenges in treating this aggressive malignancy.

## 5 Conclusions

In conclusion, hypoxia-induced downregulation of TRIM21 stabilizes ID1 and promotes pancreatic tumor progression, highlighting the TRIM21–ID1 pathway as a promising therapeutic target for pancreatic adenocarcinoma.

## Data Availability

The original contributions presented in the study are included in the article/**Supplementary Material**, further inquiries can be directed to the corresponding author/s.
