# Supplementary material for: Hypoxia promotes pancreatic adenocarcinoma progression by stabilizing ID1 via TRIM21 suppression
Source: Front Oncol. 2025 Aug 21;15:1616968. doi: 10.3389/fonc.2025.1616968 (PMC12408324; doi:10.3389/fonc.2025.1616968)
Supplement: Supplementary file 1 [file DataSheet1.pdf]

S1

A

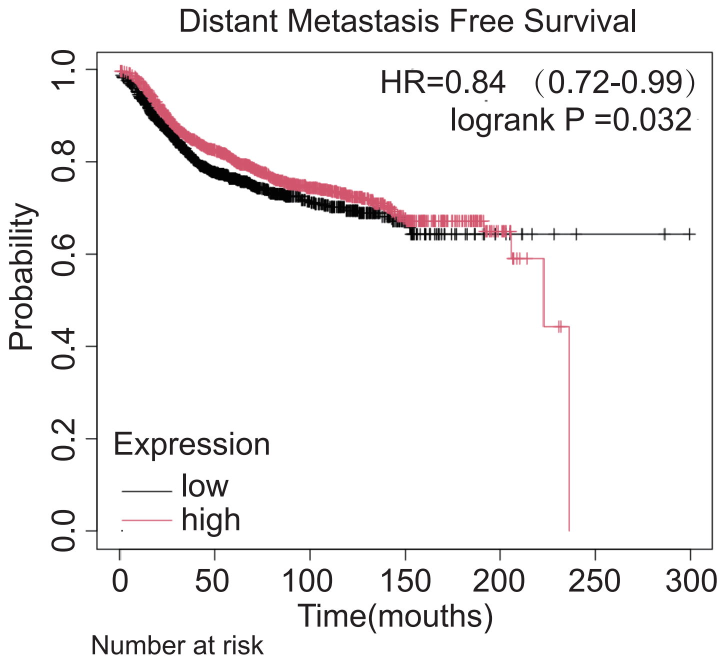

Number at risk

low	1383	707	251	52	8	2	0
high	1382	924	430	129	19	0	0

S2

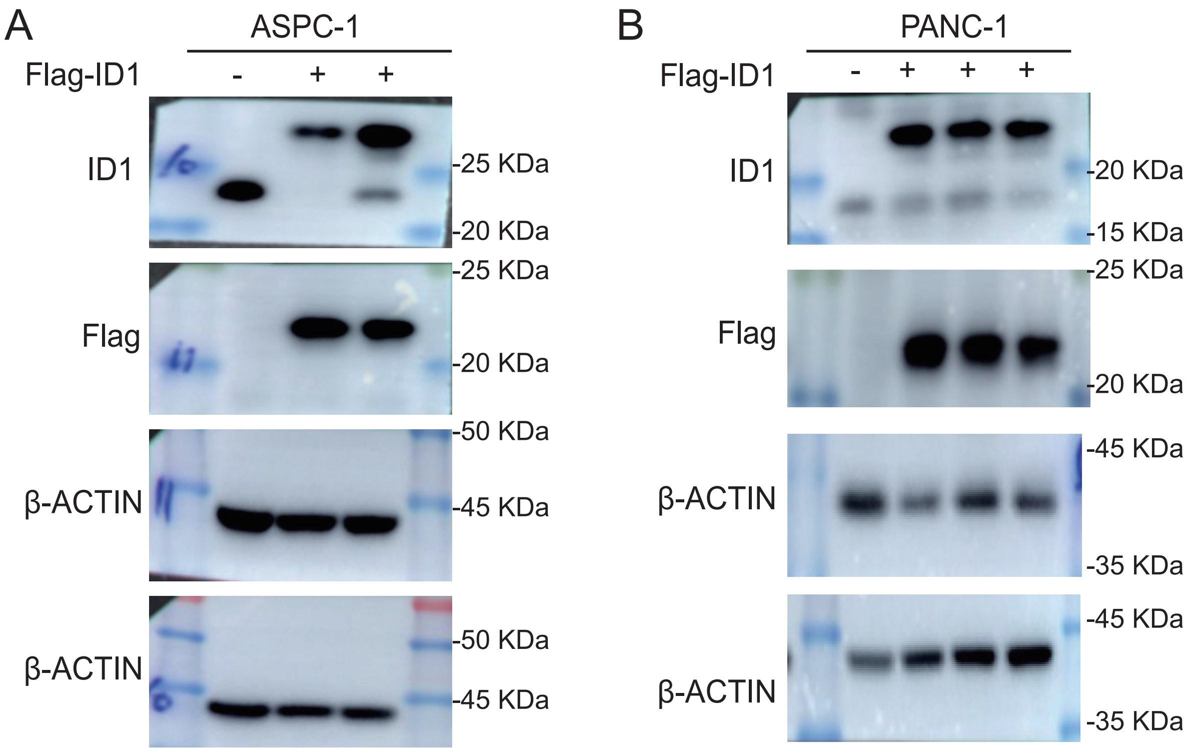
A
ASPC-1
Flag-ID1
-
+
+
ID1
25 KDa
20 KDa
Flag
25 KDa
20 KDa
β-ACTIN
50 KDa
45 KDa
β-ACTIN
50 KDa
45 KDa
B
PANC-1
Flag-ID1
-
+
+
+
ID1
20 KDa
15 KDa
Flag
25 KDa
20 KDa
β-ACTIN
45 KDa
35 KDa
β-ACTIN
45 KDa
35 KDa

S3

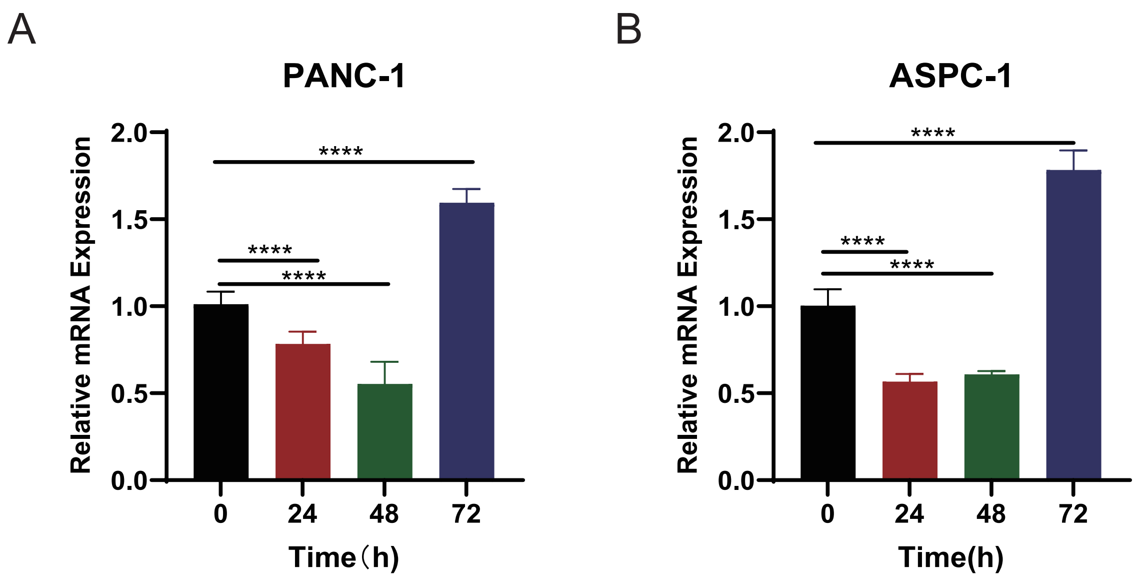
A
PANC-1
Relative mRNA Expression
2.0
1.5
1.0
0.5
0.0
****
****
****
0
24
48
72
Time（h)
B
ASPC-1
Relative mRNA Expression
2.0
1.5
1.0
0.5
0.0
****
****
****
0
24
48
72
Time(h)

S4

A

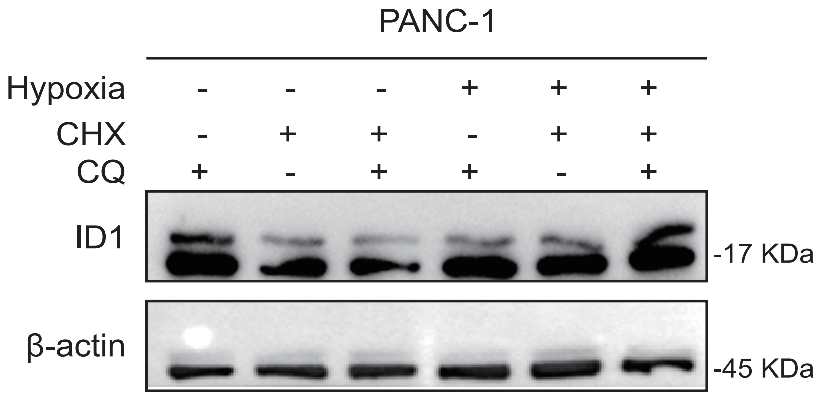

S5

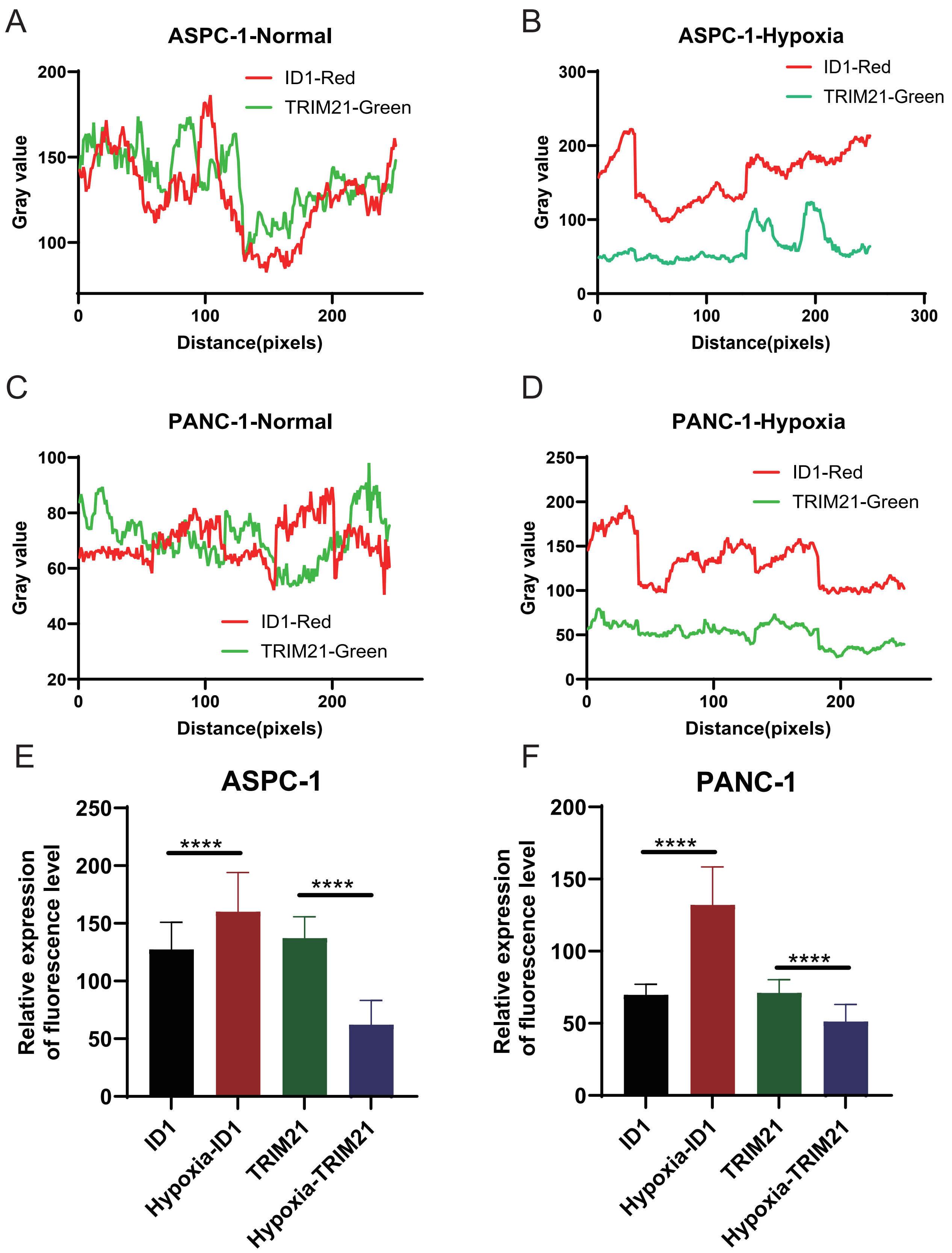
A
ASPC-1-Normal
ID1-Red
TRIM21-Green
Gray value
Distance(pixels)
B
ASPC-1-Hypoxia
ID1-Red
TRIM21-Green
Gray value
Distance(pixels)
C
PANC-1-Normal
ID1-Red
TRIM21-Green
Gray value
Distance(pixels)
D
PANC-1-Hypoxia
ID1-Red
TRIM21-Green
Gray value
Distance(pixels)
E
ASPC-1
Relative expression of fluorescence level
****
****
ID1
Hypoxia-ID1
TRIM21
Hypoxia-TRIM21
F
PANC-1
Relative expression of fluorescence level
****
****
ID1
Hypoxia-ID1
TRIM21
Hypoxia-TRIM21

S6

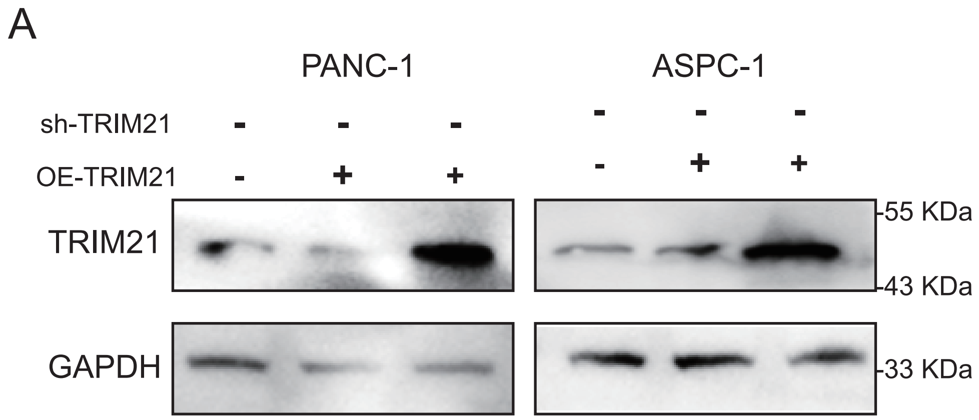
A
PANC-1
ASPC-1
sh-TRIM21
OE-TRIM21
-
+
TRIM21
GAPDH
55 KDa
43 KDa
33 KDa

Figure lengends

Figure S1. High ID1 expression correlates with poor prognosis in PAAD.
A. Kaplan–Meier survival analysis of distant metastasis-free survival (DMFS) in pancreatic adenocarcinoma (PAAD) patients stratified by high versus low ID1 expression.

Figure S2. Establishment of stable ID1-overexpressing pancreatic cancer cell lines.
A–B. ID1 was overexpressed in the indicated pancreatic cancer cell lines via transfection with Flag-ID1 plasmid. Representative Western blot analysis confirms elevated ID1 protein levels in stable ID1-overexpressing cell lines compared to controls.

Figure S3. Hypoxia induces ID1 transcription in pancreatic cancer cells.
A–B. Quantitative RT-PCR analysis of ID1 mRNA levels after 24, 48, and 72 hours of exposure to hypoxic (3% O_2) or normoxic (21% O_2) conditions in pancreatic cancer cell lines.

Figure S4. ID1 protein is stabilized by autophagy inhibition under hypoxia.
A. Western blot analysis of ID1 protein levels in cells treated with the autophagy inhibitor chloroquine (CQ, 50 μM) and protein synthesis inhibitor cycloheximide (CHX, 20 μM) for 12 hours under normoxic or hypoxic conditions.

Figure S5. Hypoxia alters TRIM21 and ID1 expression in pancreatic cancer cells.
A–F. Representative immunofluorescence images and quantitative analysis of TRIM21 and ID1 expression under normoxic and hypoxic conditions. Imaging was performed under identical acquisition settings (laser power, exposure time, and gain). Hypoxia led to a notable reduction in TRIM21 and an increase in ID1 expression. Fluorescence intensity was quantified across multiple randomly selected fields. Data are presented as mean ± SD; $P < 0.05$ by unpaired two-tailed Student's *t*-test.

Figure S6. Validation of TRIM21 overexpression and knockdown in PANC-1 and AsPC-1 cells.
A–B. Western blot analysis of TRIM21 protein levels in wild-type (WT), TRIM21-overexpressing (TRIM21-OE), and TRIM21 knockdown (shTRIM21-1) PANC-1 and

AsPC-1 cell lines. GAPDH served as the loading control. Overexpression and knockdown efficiency were confirmed in both cell lines.
